# Efficacy and Safety of the Use of Interspinous Spacers in the Treatment of Lumbar Spinal Stenosis: Mapping Gap and Scoping Review

**DOI:** 10.1111/papr.70156

**Published:** 2026-05-04

**Authors:** Juan Carlos Acevedo‐Gonzalez, Valentina Velasco‐Muñoz, Juan Jacobo Ramirez‐Triana, Danuby Adriana Buitrago‐Lopez

**Affiliations:** ^1^ Facultad de Medicina, Departamento de Neurociencias Pontificia Universidad Javeriana Bogotá Colombia; ^2^ Unidad de Neurocirugia Hospital Universitario San Ignacio Bogotá Colombia

**Keywords:** claudication, interspinous spacer, low back pain, lumbar spinal stenosis, pain, spinal stenosis, treatment

## Abstract

**Objectives:**

Low back pain is a common pathology in the general population. In people over 60 years of age, it is associated with degenerative changes that cause narrowing of the vertebral canal. Its treatment includes conservative measures and even surgery with decompression and fusion. In those patients with moderate symptoms, the use of interspinous spacers emerged as a minimally invasive therapeutic option. This scoping review seeks to map the existing literature on the use of interspinous spacers and identify knowledge gaps to clarify their real position in the stepwise approach to low back pain.

**Materials and Methods:**

This study as a scoping review, conducted in accordance with the Joanna Briggs Institute (JBI) methodology and reported following the PRISMA‐ScR guidelines. The type of synthesis chosen was descriptive and mapping‐based, allowing for the inclusion of diverse study designs (randomized controlled trials, observational studies, case series, clinical guidelines, and relevant reviews) to capture a comprehensive overview of the field. This approach was selected because the existing literature is heterogeneous in terms of intervention protocols, clinical outcomes, and follow‐up duration, and the effectiveness and indications for interspinous spacers (ISD) remain controversial. The search was extended from the inception of the databases until November 2025. The program “Rayyan” was used to collect the information and facilitate the analysis process. Each of the authors independently reviewed the summary of all the articles found and applied the following inclusion criteria: systematic review, clinical trials, observational studies, and case series. Exclusion: articles in cadavers or made in the laboratory.

**Results:**

A total of 522 articles were found in the databases consulted, of which 95 duplicate articles were eliminated. The criteria (Inclusion/Exclusion) were applied to the 427 identified articles based on the independent reading of the abstracts by each of the authors in the Rayyan platform, and 110 articles were excluded. The 317 selected articles were reviewed completely by each of the authors to finally obtain 101 articles included in the review.

**Conclusions:**

The use of ISD in the treatment of moderate LSS may be controversial. Although the clinical results seem conclusive about the usefulness in controlling symptoms, more studies are needed to compare these technologies with new surgical procedures and especially new biomechanical concepts. The efforts made to treat patients with moderate LSS appropriately should continue to be channeled into optimizing techniques.

## 1 Introduction

Low back pain (LBP) is one of the most common symptoms in the general population. It has an incidence of 1.4%–20% in the general population (44% at some point in life and 1 in 5 for a period of more than 3 months) [1, 2, 3, 4, 5, 6]. After the fifth decade of life, LBP is associated with degenerative changes inherent to aging that occur in the spine, even in asymptomatic populations (20% of people over 60 years of age and 80% of people over 70 years of age) [1, 2]. About 43% of the general population over 60 years of age may suffer from pain due to lumbar spine stenosis (LSS), being the disease that causes the most years of life with disability in the United States [2, 3, 4, 5, 6].

Patients with mild LSS can control their symptoms with adequate comprehensive (conservative) medical management schemes [3, 4, 5, 6, 7, 8]. Those with severe LSS are generally indicated for surgical decompression and fusion procedures in the context of restoration of sagittal balance [7]. But for those patients with moderate LSS, the use of second‐generation interspinous spacers (ISD) has been revived (after 2015) as an alternative treatment [9].

Since October 1958 when F.L. Knowles published the first paper of 360 patients with pain and a narrow lumbar canal, treated (since 1952) with a metal device implanted through a small incision between the lumbar spinous processes (including 6 patients with “moderate or severe spondylolisthesis”) there is concern regarding the effect of this “simple” procedure on the anterior and lateral aspects of the spine, as well as that of its effectiveness [10]. Despite reporting an improvement of more than 50% in most patients, it was not a technique that could be reproduced by other groups of specialists and only 22 years later Senegas developed the first modern interspinous device (ISD) called Wallis [11]. From that first description made by Knowles in 1958, the aspects considered even today as “novel” were already highlighted: “only the posterior spinous ligament is lengthened,” “the patient's hospital stay is just four days, and be can return to light work quite promptly,” “widening of the disc interspace,” “the bulge of the herniated disc is corrected,” “The foramen through which the spinal nerves leave the vertebral column is made larger,” “the operation corrects lordosis completely,” “completely stabilizes the unstable low back” [10].

While conventional management includes conservative (physical therapy, analgesics, epidural injections) and surgical (open decompression) options, interspinous spacers (ISD) have emerged as a minimally invasive alternative that promises to relieve symptoms with less perioperative morbidity. However, its true role within the therapeutic algorithm remains controversial, with heterogeneous evidence and variable indication criteria. This scoping review seeks to map the existing literature on the use of interspinous spacers for LSS and to identify knowledge gaps to clarify their actual position in the stepwise approach to low back pain. This revision is even more relevant after June 2025 when another of the most popular second‐generation ISDs was withdrawn from the market.

## 2 Materials and Methods

### 2.1 Literature Review

This study was designed as a scoping review, conducted in accordance with the Joanna Briggs Institute (JBI) methodology for scoping reviews and reported following the PRISMA‐ScR guidelines [12]. The aim of this synthesis was to map the extent, range, and nature of existing evidence regarding the use of interspinous spacers in the management of lumbar spinal stenosis (LSS).

The type of synthesis chosen was descriptive and mapping‐based, allowing for the inclusion of diverse study designs (randomized controlled trials, observational studies, case series, clinical guidelines, and relevant reviews) to capture a comprehensive overview of the field. This approach was selected because the existing literature is heterogeneous in terms of intervention protocols, clinical outcomes, and follow‐up duration, and the effectiveness and indications for interspinous spacers (ISD) remain controversial. The PICO question posed was to determine if in patients with LSS symptoms, regardless of the degree of severity, ISD were really more effective than other treatment options including conservative measures, decompression surgery (alone), decompression + fusion or MIS decompression techniques, in reducing pain and improving quality of life. The search words used in the databases were: “interspinous lumbar decompression Interspinous spacers,” “Interspinous fixation device,”, “Minimally Invasive Spine surgery,” “Superion,” “Vertiflex,” “Lumbar spinal stenosis,” “Neurogenic claudication,” using the logical connectors “AND,” “NOT,” and “OR”. The following databases were reviewed: Pubmed, Scopus, Embase, Ovid, EBSCO host, and Google Scholar. The search was extended from the inception of the databases until november 2025. The program “Rayyan” was used to collect the information and facilitate the analysis process [13]. This application is a platform that allows easy manipulation of the information obtained. Each of the authors independently reviewed the summary of all the articles found and applied the following inclusion criteria: systematic review, clinical trials, observational studies, and case series. Exclusion: study in cadavers or made in the laboratory. The PRISMA checklist was used to verify compliance with the established criteria [14].

### 2.2 Synthesis of Results

Data from the included studies were synthesized through a descriptive and narrative approach, following established scoping review methodology. Due to the heterogeneity in study designs, types of interspinous spacers, outcome measures, and follow‐up periods, a meta‐analysis was not performed. Instead, the synthesis mapped and categorized the characteristics of the evidence, including patient demographics, device types, surgical techniques, and comparators where available by type of research design. Clinical outcomes such as pain reduction, functional improvement, complications, and reoperation rates were summarized and presented in tables and narrative and descriptive form. Patterns of indications and contraindications were highlighted to clarify the clinical contexts in which interspinous spacers (ISD) are used, and knowledge gaps were identified to guide future research and inform their potential role within the therapeutic algorithm for older adults with lumbar spinal stenosis. From the reading of the full text of the selected articles, those that were in duplicate, narrative reviews, or studies that did not specifically deal with the use of ISD were excluded. Our synthesis was essentially descriptive and therefore we did not include a mandatory risk of bias analysis in systematic reviews and meta‐analyses.

## 3 Results

**FIGURE 1:**
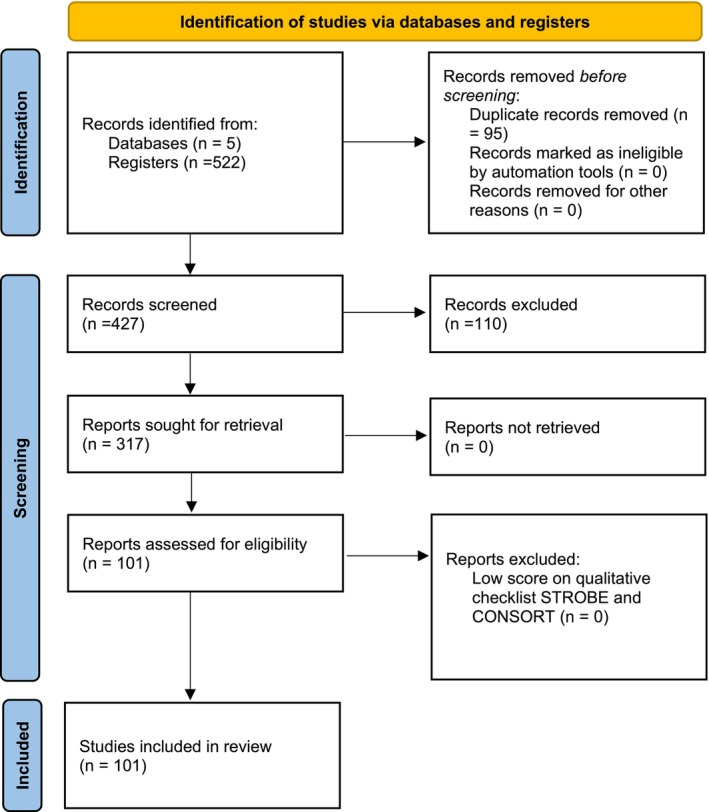
PRISMA.

A total of 522 articles were found in the databases consulted, of which 95 duplicate articles were eliminated. The criteria (Inclusion/Exclusion) were applied to the 427 identified articles based on the independent reading of the abstracts by each of the authors in the Rayyan platform and 110 articles were excluded. The 317 selected articles were reviewed completely by each of the authors to finally obtain 101 articles included in the review (Figure 1). Epidemiological and methodological data, as well as data related to the clinical results and safety of the procedures performed, were extracted on an excel table.

### 3.1 Classification of Items

**FIGURE 2:**
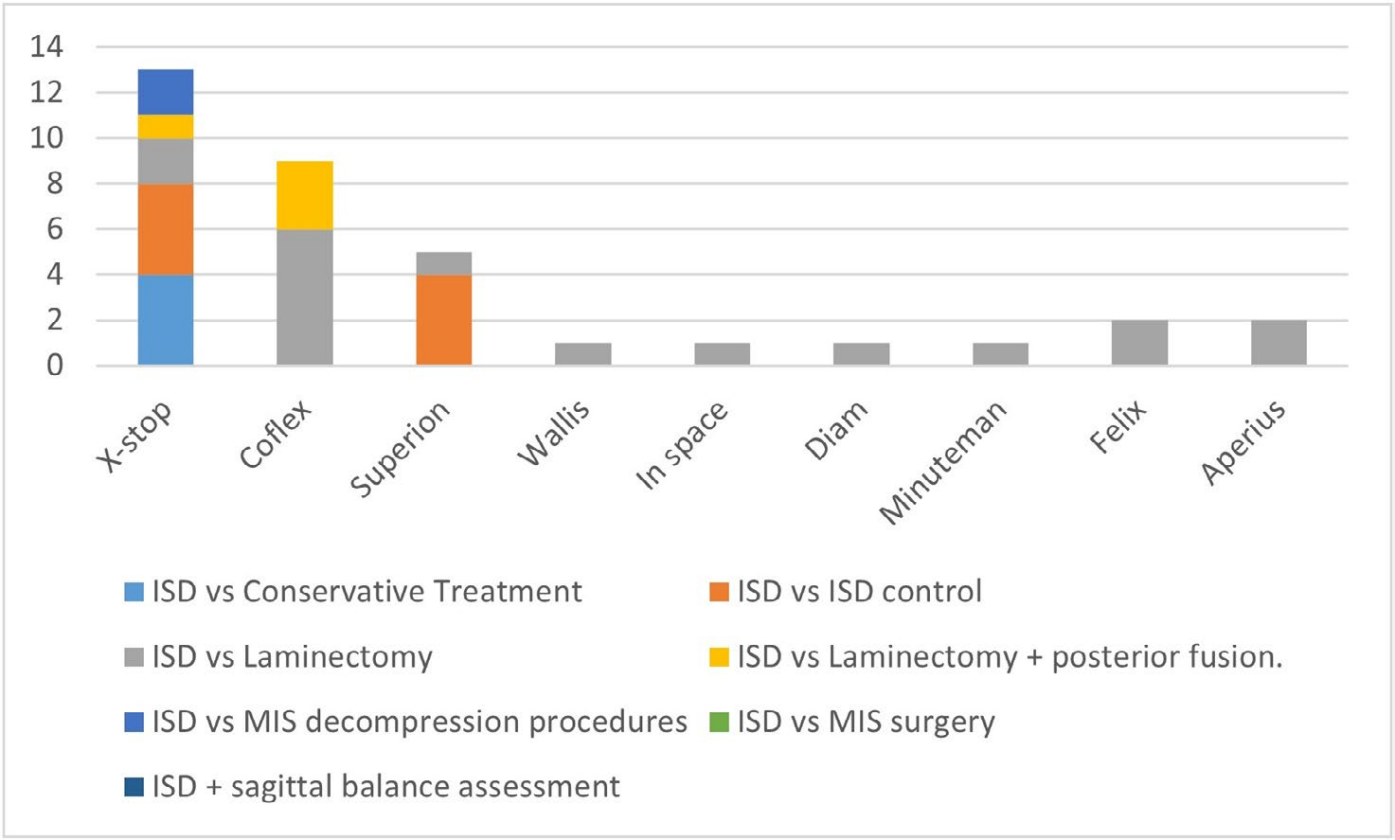
Randomized clinical trials. This figure illustrates the methodology used in the different published randomized clinical trials on ISD. It allows the number of articles to be visualized according to the type of treatment used and identifies the aspects that are least evaluated at the time of popularizing this technique.

There were 101 selected articles of which were:

- *30 randomized clinical study* [4, 9, 15, 16, 17, 18, 19, 20, 21, 22, 23, 24, 25, 26, 27, 28, 29, 30, 31, 32, 33, 34, 35, 36, 37, 38, 39, 40, 41, 42] (Figure 2 and Table 1). There were 30 selected articles, written between 2004 (Zucherman) [16] and 2024 (Baranidharan) [42]. Zucherman's (2004) study compared LSS treatment with ISD (X‐stop) vs. conservative medical treatment and a 12‐month follow‐up, observing that 59% of patients treated with ISD improved (12% with conservative treatment) [16]. The study by Baranidhasan (2024) randomized patients with LSS in a group treated with ISD (Minuteman IFD) vs. decompressive surgery without fusion, with a follow‐up of 5 years. Despite a low number of participants (48 patients), both groups showed significant improvement in leg pain, back pain, ODI disability, physical function, pain‐free walking distance, and repeated changes from position to standing position, compared to baseline and up to 24 months. There was less blood loss in the ISD group and less surgical time. A fracture of the spinous process occurred in the ISD group that spontaneously healed [42].

**TABLE 1:** Randomized clinical trials.

Randomized clinical trials	X‐stop	Coflex	Superion	Wallis	In space	Diam	Minuteman	Felix	Aperius
ISD versus Conservative Treatment	4	0	0	0	0	0	0	0	0
ISD versus ISD control	4	0	4	0	0	0	0	0	0
ISD versus Laminectomy	2	6	1	1	1	1	1	2	2
ISD versus Laminectomy + posterior fusion	1	3	0	0	0	0	0	0	0
ISD versus MIS decompression procedures	2	0	0	0	0	0	0	0	0
ISD versus MIS surgery	0	0	0	0	0	0	0	0	0
ISD + sagittal balance assessment	0	0	0	0	0	0	0	0	0
Total	13	9	5	1	1	1	1	2	2

*Note:* This table illustrates the methodology used in the different published randomized clinical trials on ISD. It allows the number of articles to be visualized according to the type of treatment used and identifies the aspects that are least evaluated at the time of popularizing this technique.

Half of the RCTs were performed before 2015 (15/30) [4, 16, 17, 18, 19, 20, 21, 22, 23, 24, 25, 26, 27, 28, 29] and most of them were performed with first‐generation ISD (Coflex, X‐stop, Diam, Wallis, Minuteman, etc.) and only one with Superion (Vertiflex) (Patel‐2014) [28]. The significance of this entry is that most of the ISDs used in these ECAs have already been withdrawn from the market. In general, most studies were conducted with X‐stop (13/30), followed by Coflex (8/30) and Superion (vertiflex) (5/30). The other RCTs were conducted with other ISDs (Wallis—1, In space—1, Diam—1, Minuteman—1, Felix −1) [4, 9, 15, 16, 17, 18, 19, 20, 21, 22, 23, 24, 25, 26, 27, 28, 29, 30, 31, 32, 33, 34, 35, 36, 37, 38, 39, 40, 41, 42].

The five studies that used Superion (vertiflex) were: Miller‐2012, Patel‐2014, Patel‐2015, Patel‐2015, and Nunley‐2016 [22, 28, 32, 33, 38]. The first by Patel (2014) was a multicenter study (80 centers in the US) of 250 patients diagnosed with moderate LSS (with an age range of 45 years or older), randomized into two groups with a 2‐year follow‐up [28]. The ISD case group (Superion/123 p) versus ISD control group (X‐stop/127 p—FDA approved since 2005). At that time, the ISD Superion (vertiflex) was in the experimental phase and was described as an ELI 6AI‐4 V titanium alloy used in other orthopedic supplies. The implants were used at one or two vertebral levels, in equal proportion in both groups. The results were similar (in both groups) regarding the improvement in neurogenic claudication (NC) at the 2‐year follow‐up [28]. Patel's second multicenter study (2015) (31 hospitals) compared Superion (vertiflex) (190 patients) versus X‐stop (201 patients) and a 3‐year follow‐up [32]. There was improvement in at least 2/3 domains on the Zurich Scale in 63 patients (63/120 patients) in group 1, compared with 49 patients (49/129 patients) in group 2. These studies allowed Superion (vertiflex) to be approved by the FDA for marketing on May 20, 2015 [9, 28, 32, 38]. The Patel‐2015 study compared to the 2014 study shows that at 36 months the percentage of revisions, reoperations and removal of the implant was higher for X‐stop (15/44 patients vs. 11/49). Patel's third study, also from 2015, uses the same study population [9, 38]. The study was published in the BMC musculoskelet disord (trial registry was NCT00692276 and the objective was to compare 2‐year clinical outcomes in patients with moderate LSS treated with superion (vertiflex) (experimental) vs. FDA‐approved x‐stop (control)) [28]. Patel's second study from 2015 was published in the journal Spine and changes the number of patients (2014 there were 123 in Superion and 127 in X‐stop, 2015 there were 190 in Superion and 201 in X‐stop) [9]. In the same 2015 article he says that sample analysis was proposed when they reached 250 included patients, 300 and 350, respectively. By 2015, 391 patients had finally been implanted (Superion 190 and control 201). Miller's (2012) study was multicenter, randomized, and controlled of 166 patients (80 patients/Superion vs. 86 patients/X‐stop) older than 45 years with moderate LSS [22]. The symptom severity score according to the Zurich scale improved more in the Superion (vertiflex) group (30%/25%), the proportion of subjects who achieved at least two out of three success criteria according to EZ was higher in Superion (75%/67%), and the VAS for axial pain went from 55 ± 27 mm to 22 ± 26 mm at 6 months with Superion versus 54 ± 29 mm to 32 ± 31 mm with X‐stop. Radiating pain went from 61 ± 26 mm to 18 ± 27 mm with Superion versus 64 ± 26 mm to 22 ± 30 mm with X‐stop [22]. The Nunley‐2016 study seeks to establish the practical clinical significance of the published findings of Superion (vertiflex) (Superion arm only) and compares them to two published laminectomy studies that included at least one of the same outcomes as the Superion trial [33]. They conclude that it is an interesting option for patients with moderate LSS who are not candidates or who do not want a decompressive surgical laminectomy.

In 4/30 studies, the use of ISD was compared with conservative medical treatment. All were made with X‐stop (withdrawn from the market in 2015). In all of them, a variable improvement (between 50% and 75% of the VAS) was observed in the group of patients with ISD, always higher than the conservative treatment group. The type of conservative medical treatment is mentioned only superficially and there is no established parameter of quality of the treatment performed, but only compliance with a period of time [16, 17, 18, 19].

There were 16/30 studies in which the use of ISD was compared with (open) surgery without fusion (laminectomy) [4, 20, 24, 26, 27, 29, 30, 33, 34, 35, 37, 38, 39, 40, 41, 42]. Only one of those studies used second‐generation spacers (Superion) and the rest (15/16) used a variety of older ISD (Coflex 6, X‐stop 2, In‐space 1, Felix 2, Wallis 1, Diam 1, Aperius 2). In general, spacers had the same or superior results as traditional surgery, but with a higher reoperation rate (ISD) and fewer adverse effects (ISD). It is important to note that the two articles published by Meyer/Le Huec belong to the “Nice trial study group,” both published in 2017, using the same study population (*Neurosurgery Journal and Global Spine Journal*) [37, 39]. Mooken's article compares ISD without bone decompression vs. laminectomy. He uses the Felix ISD and concludes that the clinical results in both groups are similar with respect to pain reduction, although with a higher number of reoperations in the ISD group [24, 30].

There were 4/30 articles that compared the use of ISD and open surgery (laminectomy) with fusion. None of the articles used second‐generation spacers (Coflex‐3, X‐stop‐1). The articles included in this item seek to demonstrate that ISD allows dynamic stabilization + decompression without the risk of generating an adjacent segment. The article by Hai [9] compared two groups of patients with degenerative spondylolisthesis l4–l5 treated with ISD (Coflex) (15p) vs. decompression + fusion with transpedicular screws (23p). They point out that the clinical results are similar in both groups and that patients in the ISD group did not present progression of spondylolisthesis at the 3‐year follow‐up, nor did they present adjacent segment syndrome. The study by Bae [36] is an RCT conducted in 21 US hospitals comparing ISD (Coflex‐196patients) versus Surgery + fusion (94patients). At 36 months, clinical improvement was achieved in 62.2% of patients in Group 1, compared to 48.9% in Group 2. The Azzazi study [21] presents 60 randomized patients in the ISD group (X‐stop) versus decompression surgery + fusion group. All patients had G1 spondylolisthesis. Follow‐up was done for up to 24 months and better outcomes were observed in the ISD group with fewer complications. The Davis study [23] presents 322 patients treated at 21 centers in the U.S. between 2006 and 2008. They evaluated the cohort of patients with G1 spondylolisthesis (ISD—99patients vs. Surgery + fusion 51patients). They were randomized 2:1 and followed up for 2 years. They conclude that the use of ISD allows similar results in patients with low‐grade spondylolisthesis, but with a much higher percentage of reoperation. They point out that the ISD group presented fewer modifications in the adjacent vertebral segment compared to the fusion group [9, 21, 23, 36].

**FIGURE 3:**
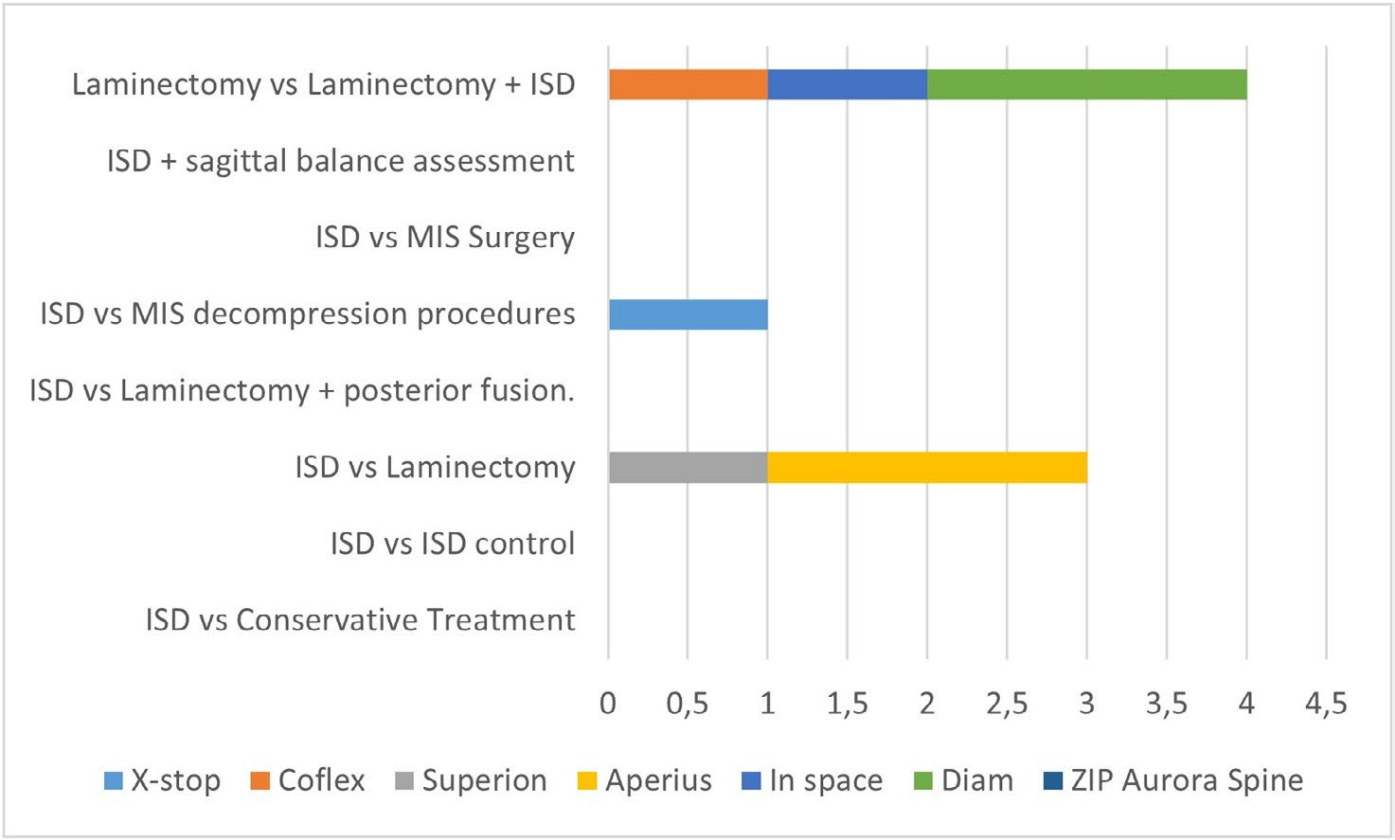
Prospective clinical trials. This figure illustrates the methodology used in the different published prospective studies on ISD. It allows the number of articles to be visualized according to the type of treatment used and identifies the aspects that are least evaluated at the time of popularizing this technique.

**FIGURE 4:**
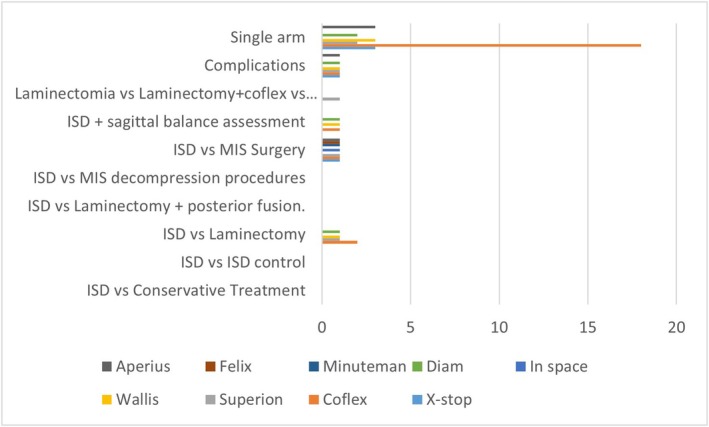
Retrospective studies. This figure illustrates the methodology used in the different published retrospective studies on ISD. It allows the number of articles to be visualized according to the type of treatment used and identifies the aspects that are least evaluated at the time of popularizing this technique.

Among the other included studies, we noted 2/30 that compared X‐stop (ISD withdrawn in 2015) and minimally invasive decompression surgery [26, 31]. There were 4/30 studies that directly compared ISD (X‐stop) versus ISD (Superion). These are the same as those already analyzed in the item of ECAS carried out with Superion (three articles by Patel and 1 article by Miller) [9, 28, 32, 33].

b 13 prospectives study [43, 44, 45, 46, 47, 48, 49, 50, 51, 52, 53, 54, 55] (Figure 3 and Table 2). There were 13 prospective studies written between 2010 and 2023. Only 3/13 used second‐generation spacers (Superion‐2 and ZIP (Aurora Spine)‐1) [45, 52, 55], and 10/13 other ISDs (Coflex‐3, X‐stop‐1, Aperius‐3, Diam‐2, In‐space‐1). Among these studies, it is important to mention the one by Staats [54] that compares the analysis of the records of complications in patients treated with minimally invasive decompression technique versus patients treated with ISD (different types). It concludes that the results are similar with respect to reinterventions, but with a lower percentage of Medicare claims for the MIS decompression group [54]. The prospective study by Bini [46] measuring efficacy and safety in patients treated with ISD (Superion) was one of the first published for this device in 2011 and pointed to its usefulness in the treatment of patients with moderate LSS (121 patients). Some of these prospective studies compare two treatment groups (ISD vs. Surgery) without randomization (Staats—ISD Minuteman vs. Surgery MIS, Ghany—Surgery vs. Surgery + Coflex, Beyer—ISD Aperius vs. Surgery, Sobottke—ISD Aperius vs. Surgery, Galarza—Microdiscectomy vs. Microdiscectomy + ISD (23 Diam, 22 Intraspine), Ryu—Decompression Surgery vs. Decompression + Diam) with similar results regarding pain improvement but with less surgical time for the ISD group. less bleeding and a higher percentage of reoperation [44, 46, 48, 50, 54]. Galarza's study compares 92 patients with herniated discs at the L5‐S1 level treated with microdiscectomy (Group 1) vs. microdiscectomy + ISD (Group 2). This is one of the few studies evaluating ISD at the L5‐S1 vertebral level. Both groups showed clinical improvement, but using the MacNab improvement criteria, Group 2 was superior (44% vs. 80%) [48].
c 44 Retrospective study [1, 5, 56, 57, 58, 59, 60, 61, 62, 63, 64, 65, 66, 67, 68, 69, 70, 71, 72, 73, 74, 75, 76, 77, 78, 79, 80, 81, 82, 83, 84, 85, 86, 87, 88, 89, 90, 91, 92, 93, 94, 95, 96](Figure 4 and Table 3). There were 44 studies published between 2009 and 2025. Most of them exclusively included the Coflex ISD (23/44), 5 of them X‐stop and 6/44 ISD Superion (second generation) [1, 67, 82, 86, 96].

**TABLE 2:** Prospective clinical trials.

Prospective studies	X‐stop	Coflex	Superion	Aperius	In space	Diam	ZIP Aurora Spine
ISD versus Conservative Treatment	0	0	0	0	0	0	0
ISD versus ISD control	0	0	0	0	0	0	0
ISD versus Laminectomy	0	0	1	2	0	0	0
ISD versus Laminectomy + posterior fusion.	0	0	0	0	0	0	0
ISD versus MIS decompression procedures	1	0	0	0	0	0	0
ISD versus MIS Surgery	0	0	0	0	0	0	0
ISD + sagittal balance assessment	0	0	0	0	0	0	0
Laminectomy versus Laminectomy + ISD	0	1	0	0	1	2	0
ISD Single Arm	0	2	1	1	0	0	1
#	1	3	2	3	1	2	1

*Note:* This table illustrates the methodology used in the different published prospective studies on ISD. It allows the number of articles to be visualized according to the type of treatment used and identifies the aspects that are least evaluated at the time of popularizing this technique.

**TABLE 3:** Retrospective studies.

Retrospective studies	X‐stop	Coflex	Superion	Wallis	In space	Diam	Minuteman	Felix	Aperius
ISD versus Conservative Treatment	0	0	0	0	0	0	0	0	0
ISD versus ISD control	0	0	0	0	0	0	0	0	0
ISD versus Laminectomy	0	2	1	1	0	1	0	0	0
ISD versus Laminectomy + posterior fusion.	0	0	0	0	0	0	0	0	0
ISD versus MIS decompression procedures	0	0	0	0	0	0	0	0	0
ISD versus MIS Surgery	1	1	1	0	1	0	1	1	1
ISD + sagittal balance assessment	0	1	0	1	0	1	0	0	0
Laminectomia versus Laminectomy+coflex versus Superion	0	0	1	0	0	0	0	0	0
Complications	1	1	1	1	0	1	0	0	1
Single arm	3	18	2	3	0	2	0	0	3
#	5	23	6	6	1	5	1	1	5

*Note:* This table illustrates the methodology used in the different published retrospective studies on ISD. It allows the number of articles to be visualized according to the type of treatment used and identifies the aspects that are least evaluated at the time of popularizing this technique.

The articles that used ISD Superion (vertiflex) were 6/44 (Shabbat‐2011, Nunley‐2017, Nunley‐2018, Tekmyster‐2019, Hartman‐2019, Nelson‐2025) [1, 67, 83, 86, 87, 96]. The Shabbat study [67] is one of the first studies of the ISD Superion (vertiflex) with 53 patients with moderate LSS and implanted with follow‐up of 2 years. Axial pain decreased from 8.9 ± 1.4 mm to 4.1 ± 3.4 mm postoperatively. Pain in the extremities decreased from 8.7 ± 1.9 mm to 4.1 ± 3.2 mm postoperatively. The studies by Nunley (2017 and 2018) [83, 86] include the same population of patients treated with ISD Superion. In the first of them, they evaluate opioid consumption and its reduction after treatment, with a follow‐up of 5 years. 50% (94 of 190 patients) were using opioids prior to treatment and this dropped to only 7.5% (8 of 107 patients) after treatment. Nunley's second study presents clinical results at 5 years of follow‐up with ISD Superion treatment. At 5 years, 74/88 patients had clinical improvement in at least 2/3 of the domains of the Zurich scale. The success rate of improvement with respect to leg pain was 80% (68/85 patients) and 65% for low back pain (55/85 patients). 25% of patients had required reoperation, revision and/or supplementary fixation after 5 years. The Tekmyster study [87] evaluates the real‐world use of ISD Superion after commercialization. It includes 1426 patients treated by 316 medical specialists (implanters) at 86 medical centers in the U.S. who use the device. A telephone control of the clinical results was carried out retrospectively, and the following were evidenced: Greater clinical improvement of leg pain than that of the lumbar region at 12 months of follow‐up (VAS leg pain went from 76.6 ± 22.4 mm to 30.4 ± 34.6 mm vs. VAS in the lumbar region went from 76.8 ± 22.2 mm to 39.9 ± 32.3 mm). The percentage of reoperation was 3.6%. Hartman's study [1] has a title that seems to indicate that it is specific to ISD Superion but compares three treatment groups retrospectively. A group of patients (45p) treated with laminectomy with or without fusion vs. a group of patients (28p) treated with laminectomy + coflex and a group of patients (13p) treated with Superion (vertiflex). Among those patients, they point to one who required ISD Coflex at the L4‐L5 level and 20 months later was treated with ISD Superion at the adjacent L3‐L4 level. Although it makes a general analysis of results, it really describes in a narrative way the experience with the three techniques.

The study by Tamburrelli [63] is very interesting because it retrospectively analyzes the poor results of a series of 19 patients implanted with ISD (11 X‐stop, 5 Diam, 3 Coflex, 2 Bacjak, 2 Wallis, 1 Aperius, 1 Viking and 1 Superion). He identified that the most common cause of ISD failure was an erroneous indication. It is contraindicated for patients with severe LSS, in patients with severe deformity (scoliosis) or instability. They identified technical errors that are difficult to diagnose in devices that are not radiolucent. Superion, X‐stop, and Aperius have the advantage of properly checking the position of the ISD with Rx.

We mention the only 2 articles in this review that comprehensively analyze the modifications in sagittal balance associated with ISD. Wang's study [91] was published in 2023 and presents the analysis of 89 patients treated with ISD Coflex and decompression + fusion. For the analysis, patients were divided into three groups (G1: L4‐L5 ISD + L5‐S1 Fusion, G2: L3‐L4 ISD + L4‐S1 Fusion, G3: L2‐L3 ISD + L3‐S1 Fusion). Local sagittal parameters (angle in the implanted segment, angle of the intervertebral disc, foramen height and disc height) and global sagittal parameters (lumbar lordosis, angle of the fused segment, sacral slope, pelvic tilt, pelvic incidence, vertical sagittal axis) were evaluated for each patient and correlated with clinical outcomes (ODI and VAS). They conclude that the placement of ISD in the segment above fusion produces a temporary loss of local lordosis, especially in the lower lumbar segment with a realignment of the intervertebral space in the medium‐term follow‐up. The ISD Coflex works as a device that restricts the range of motion of the implanted level by exerting an elastic cushioning process.

The study by Korovesis [85] was published in 2018 and presents the retrospective analysis of 55 patients treated with decompression + fusion, divided into three groups (G1: ISD PEEK—Wallis (17 patients), G2: ISD Silicon—DIAM (18 patients), G3: Control No ISD (20 patients)). Spinopelvic balance was measured with SVA, T12‐S1 (LL) Lordosis, (SS) sacral slope, (PT) pelvic tilt, (PI) pelvic incidence, and upper disc height. They identified that SS and the height of the overlying disc increased in the postoperative period in a compensatory manner. They found that 6/17 in G1, 4/18 in G2 and 5/20 in G3, showed radiological progression of degenerative changes in the upper disc to the implant and three patients developed adjacent segment syndrome (2/17 in G1, 1/18 in G2 and 2/20 in G3).

d *13 Metaanalisis study* [3, 97, 98, 99, 100, 101, 102, 103, 104, 105, 106, 107, 108]. There were 12 studies conducted between 2014 and 2024, most of which (10/12) were published after 2015. The study by Wu (2014) seeks to compare clinical outcomes of ISD (X.stop, Coflex, Diam, and Aperius) with decompressive surgery alone in patients with LSS [97]. The search period runs until August 2013, including RCTs and prospective studies of at least 30 patients and 12‐month follow‐up. They included 2 RCTs and 3 prospective studies for a total of 204 patients in the ISD group and 217 in the surgery group. They conclude that the benefits are similar but the percentage of reoperation in the ISD group is higher [97]. The study by Han (2024) seeks to evaluate the efficacy and safety of ISD in older patients with LSS [3]. The search period lasts until October 2023. They included 5 RCTs involving 555 patients. It concludes that the clinical results in patients treated with ISD are not superior to decompressive surgery but have a lower reoperation rate [3]. Among the other meta‐analyses, we highlight that there were 5 studies that compared ISD vs. surgery (decompression) (Wu, Zhu, Han, Xin, Zhao) [3, 97, 99, 107, 108], 2 ISD versus Fusion (Li, Li) [100, 106], 1 Superion versus X‐stop (Zhao) [103], 1 Surgery alone vs. fusion + coflex (Fan) [104], 2 that compares ISD versus conservative treatment versus surgery versus ISD + fusion (Li, Liang) [101, 105], 1 surgery versus fusion versus ISD (Zhang) [105]. RCTs included in all meta‐analyses are analyzed in our review.
e *1 Systematic review* [3, 108]. These were two systematic review studies. Yaghoubi's study is a cost‐effectiveness analysis between ISD (Coflex and X‐stop) versus Surgery. They conclude that the ISD X‐stop (already withdrawn from the market) is the most profitable [3].

## 4 DISCUSION

The historical evolution of the use of ISDs in the treatment of LSS has been notoriously irregular [10, 11, 109]. It was 22 years between Knowles' first description of an ISD in 1958 and the first modern device developed by Senegas in 1980 (Wallis) [10, 11, 110]. It had years of exponential growth (Coflex, Diam) and then fell into periods of technological stagnation and withdrawal from the market by the producing companies (X‐stop) [111]. At the same time, in 2015 (in parallel), the first second‐generation ISD (Superion) received approval for use in humans by the FDA and the comparator ISD (X‐stop), which had shown similar clinical results in different studies was withdrawn [9, 28, 32]. Recently (June 2025) the ISD Superion (Vertiflex) was withdrawn from the market, as happened 10 years ago with its X‐stop comparator at that time.

The purpose of using these devices (ISD) in the treatment of LSS has not changed since Knowles' first article, as have the clinical arguments, advantages, and possible mechanisms of action [10]. Based on the increase in the incidence (and prevalence) of low back pain and neurogenic claudication in patients over 60 years of age due to LSS, there is still great interest in offering minimally invasive treatments that improve symptoms, restoring activity and functionality quickly, with minimal risks during implantation. These same aspects considered as novel each time a new input (ISD) is offered were the same arguments used in Knowles' classic article [10]. It is true that there is a group of patients with moderate LSS, who respond little to conservative treatments, but in whom the severity of the symptoms does not justify fusion surgical procedures [7]. That is why the proposal of a minimally invasive implant approved even to be implanted in ambulatory care centers will always seem very interesting. However, this need identified in a specific population (moderate LSS) should be oriented above all to improve conservative treatments with specific goals and to popularize MIS decompression and fusion techniques [3, 109, 110].

**FIGURE 5:**
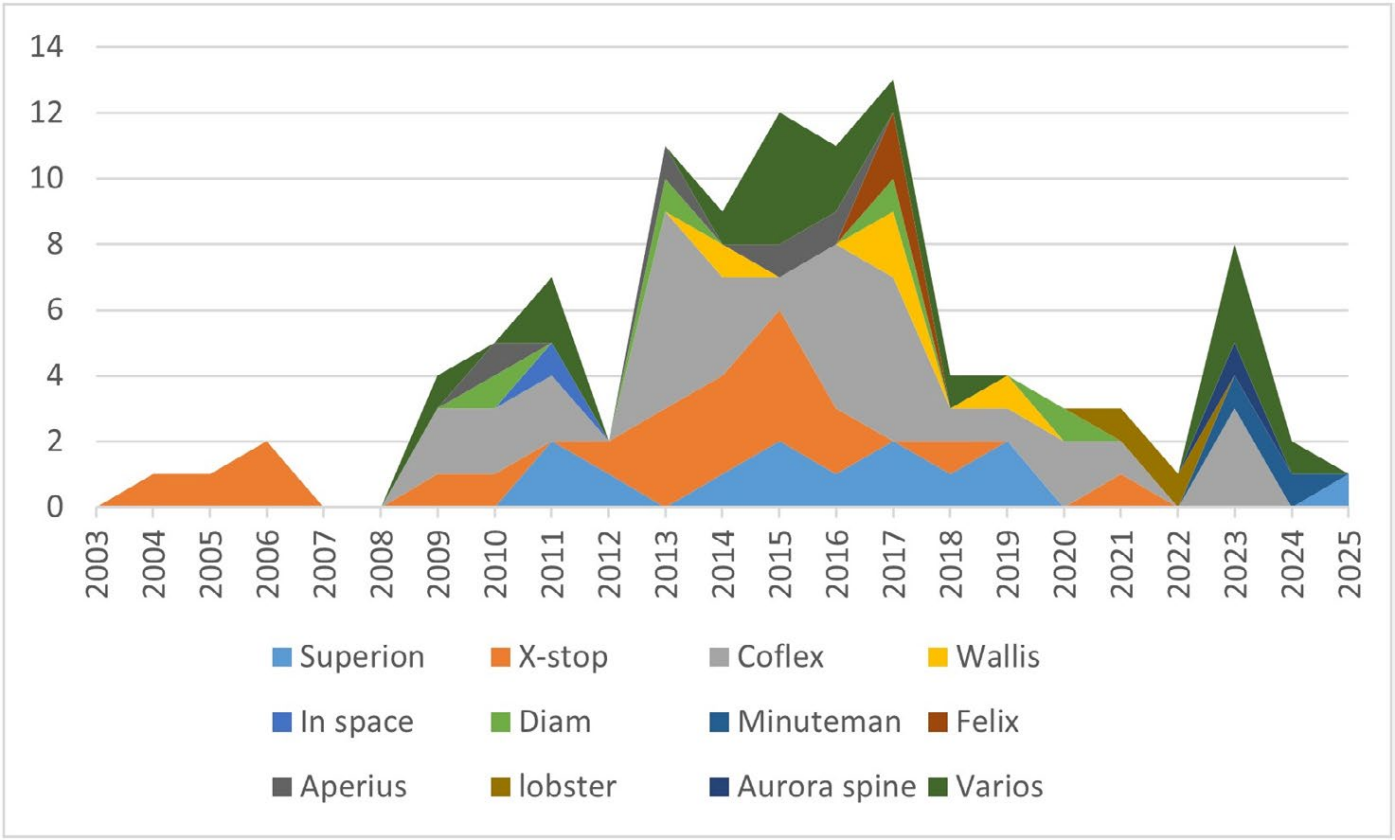
Global timeline. Distribution over the years of the number of articles published related to the type of ISD implanted. It allows you to visualize the dimension of the global research related to this type of input and the detailed dimension of each of the different ISDs.

**FIGURE 6:**
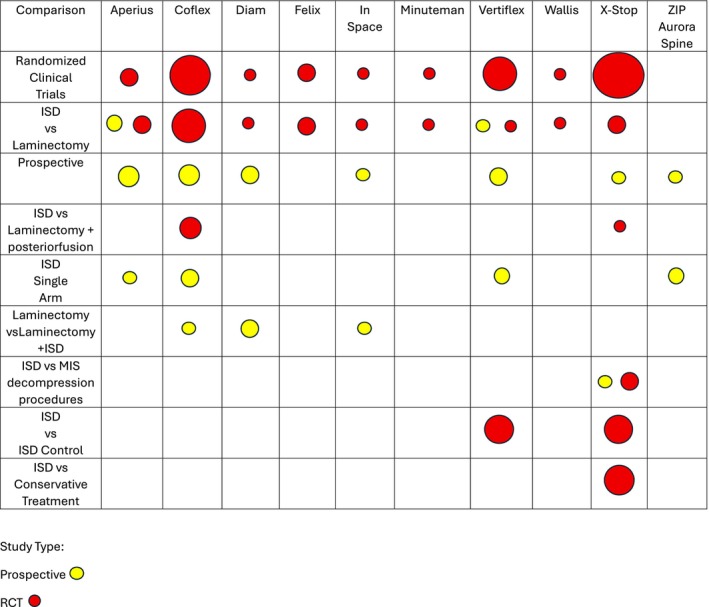
Mapping the evidence. Illustration of the distribution of published randomized clinical trials and prospective studies on ISD. It differs according to the type of DSI studied and the control or comparator group.

This systematic review allowed us to identify relevant aspects that help to understand the irregular evolution of the use of ISD in the treatment of LSS. We analyze these points below:

- Published studies: Although there were 101 studies included in this review, only 30/101 (30%) were RCTs published over a 21‐year period (Zucherman—2004, Baranidhasan—2024) [16, 42]. Among those, half were performed prior to FDA approval of second‐generation ISDs and only 5/100 (5%) used Superion (vertiflex). This means that surprisingly few studies confirm the usefulness of this procedure and some of them use the same implanted population presenting short‐, medium‐ and long‐term follow‐up [22, 28, 32, 33, 38]. We also identified other aspects that limit the possibility of drawing categorical conclusions: there are only 4 RCTs comparing ISD vs. conservative medical treatment [16, 17, 18, 19], only 4/30 RCTs comparing ISD vs. surgery + fusion [9, 21, 23, 36], only 2/101 studies measuring spinopelvic parameters related to sagittal balance [85, 91], only 1/101 studies analyzing a population of patients who failed with ISD by evaluating its possible causes [63], there is no study related to the recall of most first‐generation ISD and some second‐generation ISD. (Figures 5 and 6, Table 4).
- Degenerative changes in the lumbar spine: A relevant aspect in the analysis of the results of invasive treatments in the lumbar spine is the interpretation of radiological findings and their integration into the clinical analysis. LSS is defined radiologically as the 25%–50% reduction of the central vertebral canal and foramina compared to adjacent levels [112, 113, 114]. In CT scan of the spine a diameter of less than 10 mm is considered an absolute stenosis and < 13 mm is relative [87, 113, 115]. Degenerative changes of the lumbar spine are inherent to aging, as occurs in any tissue of the body, but they are more relevant in those structures that are subjected to biomechanical phenomena of support, as occurs in the lumbar region. In this region, these degenerative changes may be adaptive to the aging processes of the pelvis, hips, and the rest of the spine, without necessarily being pathological [116, 117]. However, it should be remembered that LSS can be seen in 20% of people over 60 years of age and in 80% of those over 70 years of age, even in those asymptomatic individuals [118]. This implies that the radiological LSS + pain duo does not necessarily justify invasive treatment of the lumbar spine, and other possible causes of the symptoms must be ruled out. This analysis is of vital importance when trying to understand the exponential increase in the implementation of ISD and even spinal surgery in recent years (in 1980 1.3/100,000 inhabitants, in 2000 19.9/100,000) [17, 24, 30, 33, 46].
- Clinical diagnosis of low back pain and neurogenic claudication. It is essential to clearly define the clinical diagnostic criteria for LSS to be able to correlate them adequately with radiological images and to make accurate diagnoses in order to propose correct invasive treatments. The characteristic symptomatic LSS produces Levy's syndrome: pain in the extremities, numbness, and subjective weakness in the back and legs with difficulty walking. Pain and paresthesias that appear when walking and standing for a long time improve when sitting or lying down. Improvement in pain at rest and with flexion of the spine in a standing position (worsening with extension) is observed in up to 82% of patients with LSS. However, depending on whether it is concentric, central, and/or lateral narrowing, the symptoms may vary. Likewise, if it is accompanied by spondylolisthesis with signs of instability. In LSS, low back pain (without claudication) has a diagnostic sensitivity of 93% and only a specificity of 46%. Irradiation due to Levy syndrome (82% of patients) must be differentiated from root irradiation due to radiculopathy and foraminal narrowing, which has a sensitivity of 88% and specificity of only 34%. The Romberg maneuver is positive even though it is frequently not taken into account (diagnostic specificity of 90% and 40% sensitivity), as well as paresis of the extensor toes (sensitivity of 50% and specificity of 80%) [7, 39, 93, 97, 112, 115, 119].
- Zurich Scale: It was developed in 1996 by Gerald Stucki and is also known as the Swiss LSS measure or the Brigham LSS questionnaire [118, 120, 121, 122]. It was specifically described to evaluate the clinical outcome of patients with LSS treated with decompression surgery. It has 3 domains including symptom severity (impaired balance, pain, and neuroischemic symptoms in lower limbs), physical function (walking mobility), and patient satisfaction (pain relief, ability to walk, and ability to perform household or work tasks). Most articles mention and use this scale to assess treatment outcomes. They consider as a minimum for a good result the improvement in at least 2/3 of domains from an arithmetic means where the higher the value, the worse the result. However, we consider that to evaluate the efficacy of invasive treatment, an improvement in all three domains should be expected [118, 120, 121, 122].
- Conservative medical treatment. The definition of conservative measures for the treatment of patients with mild or moderate LSS should be specified in detail. Most studies consider only a period of 3–6 months as the only requirement to consider conservative treatment to be carried out, without describing the specific goals and the type of conservative measure taken. Only 4/30 RCTs compared ISD vs. conservative treatment. Defining a conservative medical treatment should include specific goals such as modification in lifestyle habits, weight control, reduction of obesity, improvement of osteoporosis, improvement of sarcopenia, evaluation of physical activity and sedentary lifestyle, emotional aspects, depression, insomnia, percutaneous procedures done under a minimum standard, etc.
- Decompression and/or fusion surgery. Open surgical techniques for the treatment of LSS have evolved in a very satisfactory way and are not the same or with the same principles as those performed 15 or 20 years ago. Most studies compare the efficacy and safety of ISD with traditional laminectomy techniques that, considering current advances in spine surgery, are performed only exceptionally. This also applies to subsequent fusion techniques, which have been modified. Recent studies comparing ISD with minimally invasive fusion surgery are needed.
- Sagittal balance: Only 2/101 studies comprehensively evaluate changes in sagittal balance after ISD treatment. Both studies are retrospective. The great advances in the knowledge of the painful pathology of the lumbar spine and the aging processes are associated with the concepts related to sagittal balance. Local degenerative changes, including LSS, can only be understood in the context of their relationship with the lumbar spine, with the sacroiliac joints, the pelvis, the hips and of course the dorsal spine, cervical spine and the position of the head with the gaze to the horizon. Wanting to simplify these recognized pathophysiological mechanisms and treat pain as a segmental pathology when it is global can limit the results and prevent benefits for the patient. ISD is understood as a system that limits movement in the implanted segment and indirectly influences the diameter of the canal. However, more studies are needed to understand the direct influence of ISD in limiting extension, distracting foramina and intervertebral space on adjacent segments, on lumbar lordosis, on neighboring joints, and on sagittal balance. Pathological biomechanical forces are not canceled out; they are just displaced. Even the failures reported in ISD systems with displacements and fractures of the material could be explained and understood by the modification of biomechanical forces [117, 118, 120, 121].

**TABLE 4:** Global timeline.

	Vertiflex	X‐stop	Coflex	Wallis	In space	Diam	Minuteman	Felix	Aperius	Lobster	Aurora spine	Varios	RS + m
#	13	21	34	4	1	4	2	2	4	2	1	16	14
2003	0	0	0	0	0	0	0	0	0	0	0	0	0
2004	0	1	0	0	0	0	0	0	0	0	0	0	0
2005	0	1	0	0	0	0	0	0	0	0	0	0	0
2006	0	2	0	0	0	0	0	0	0	0	0	0	0
2007	0	0	0	0	0	0	0	0	0	0	0	0	0
2008	0	0	0	0	0	0	0	0	0	0	0	0	0
2009	0	1	2	0	0	0	0	0	0	0	0	1	0
2010	0	1	2	0	0	1	0	0	1	0	0	0	0
2011	2	0	2	0	1	0	0	0	0	0	0	2	0
2012	1	1	0	0	0	0	0	0	0	0	0	0	0
2013	0	3	6	0	0	1	0	0	1	0	0	0	0
2014	1	3	3	1	0	0	0	0	0	0	0	1	1
2015	2	4	1	0	0	0	0	0	1	0	0	4	1
2016	1	2	5	0	0	0	0	0	1	0	0	2	1
2017	2	0	5	2	0	1	0	2	0	0	0	1	3
2018	1	1	1	0	0	0	0	0	0	0	0	1	1
2019	2	0	1	1	0	0	0	0	0	0	0	0	0
2020	0	0	2	0	0	1	0	0	0	0	0	0	2
2021	0	1	1	0	0	0	0	0	0	1	0	0	0
2022	0	0	0	0	0	0	0	0	0	1	0	0	1
2023	0	0	3	0	0	0	1	0	0	0	1	3	2
2024	0	0	0	0	0	0	1	0	0	0	0	1	2
2025	1	0	0	0	0	0	0	0	0	0	0	0	0

*Note:* Distribution over the years of the number of articles published related to the type of ISD implanted. It allows you to visualize the dimension of the global research related to this type of input and the detailed dimension of each of the different ISDs.

## 5 Conclusions

The use of ISDs in the treatment of moderate LSS may be controversial. Although the clinical results seem conclusive about the usefulness in controlling symptoms, more studies are needed to compare these technologies with new surgical procedures and especially new biomechanical concepts. The efforts made to treat patients with moderate LSS appropriately should continue to be channeled into optimizing techniques.

## Author Contributions

All authors (Acevedo‐Gonzalez, Velasco‐Muñoz, Ramirez‐Triana, Buitrago‐Lopez) contributed substantially to the study concept and design, acquisition, analysis, interpretation, and material preparation. The first draft of the manuscript was written by ACEVEDO‐GONZALEZ Juan Carlos, and all authors commented on previous versions of the manuscript. All authors read and approved the final manuscript.

## Funding

The authors have nothing to report.

## Ethics Statement

The authors have nothing to report.

## Consent

The authors have nothing to report.

## Conflicts of Interest

The authors declare no conflicts of interest.

## Data Availability

Data sharing not applicable to this article as no datasets were generated or analyzed during the current study.
